# The Association between Bundled Payment Participation and Changes in Medical Episode Outcomes among High-Risk Patients

**DOI:** 10.3390/healthcare10122510

**Published:** 2022-12-12

**Authors:** Joshua M. Liao, Erkuan Wang, Ulysses Isidro, Jingsan Zhu, Deborah S. Cousins, Amol S. Navathe

**Affiliations:** 1 Department of Medicine, School of Medicine, University of Washington, Seattle, WA 98195, USA; 2 Leonard Davis Institute of Health Economics, University of Pennsylvania, Philadelphia, PA 19104, USA; 3 Department of Medical Ethics and Health Policy, Perelman School of Medicine, University of Pennsylvania, Philadelphia, PA 19104, USA; 4 Brigham & Women’s Hospital, Boston, MA 02115, USA; 5 Corporal Michael J. Crescenz VA Medical Center, Philadelphia, PA 19104, USA

**Keywords:** health policy, health care payment

## Abstract

Background: Bundled payments for medical conditions are associated with stable quality and savings through shorter skilled nursing facility (SNF) length of stay. However, effects among clinically higher-risk patients remain unknown. Objective: To evaluate whether the association between participation in bundled payments for medical conditions and episode outcomes differed for clinically high-risk versus other patients. Design: Retrospective difference-in-differences analysis; Participants: 471,421 Medicare patients hospitalized at bundled payment and propensity-matched non-participating hospitals. Exposures were 5 measures of clinically high-risk groups: advanced age (>85 years old), high case-mix, disabled, frail, and prior institutional post-acute care provider utilization. Main Measures: Primary outcomes were SNF length of stay and 90-day unplanned readmissions. Secondary outcomes included quality, utilization, and spending measures. Key Results: SNF length of stay was differentially lower among frail patients (aDID −0.4 days versus non-frail patients, 95% CI −0.8 to −0.1 days), patients with advanced age (aDID −0.8 days versus younger patients, 95% CI −1.2 to −0.3 days), and those with prior institutional post-acute care provider utilization (aDID −1.1 days versus patients without prior utilization, 95% CI −1.6 to −0.6 days), compared to non-frail, younger, and patients without prior utilization, respectively. BPCI participation was also associated with differentially greater SNF LOS among disabled patients (aDID 0.8 days versus non-disabled patients, 95% CI 0.4 to 1.2 days, *p* < 0.001). Bundled payment participation was not associated with differential changes in readmissions in any high-risk group but was associated with changes in secondary outcomes for some groups. Conclusions: Changes under medical bundles affected, but did not indiscriminately apply to, high-risk patient groups.

## 1. Introduction

A number of different countries have used bundled payments as a form of health care payment [1,2,3,4,5]. In the US, one of the largest national efforts to date has been the Bundled Payments for Care Improvement (BPCI) Initiative, a five-year program that engaged over 1000 organizations to accept bundled payments via participation options (called “models”) for up to 48 different clinical episodes [6]. This program was implemented within Medicare, the national public insurance scheme for older Americans (65 years of age and older) and those with certain disabilities (e.g., end stage renal disease).

Participation in Medicare’s BPCI program was the highest for Model 2, in which bundles were defined for episodes spanning from hospitalization for a given condition or procedure through up to 90 days of post-discharge care. Traditionally (i.e., outside of the BPCI program context), hospital and post-discharge services in the US have been paid separately, with no designated accountable clinical groups or entities for episode spending quality or spending.

BPCI sought to change these dynamics by designating participating hospitals as accountable entities for total spending and quality outcomes across the episode of care. In exchange for accepting this accountability, participating hospitals were eligible for additional financial bonuses or penalties based on their ability to maintain or improve quality, as well as contain spending compared to historical amounts. Ultimately, the goal of BPCI was to increase the value of care by containing health care spending while maintaining or improving the quality of care.

Hospital participation in BPCI has been associated with stable quality and cost savings driven by shorter length of stay at skilled nursing facilities (SNFs) among patients hospitalized for common medical conditions [7]. However, it remains unknown how the changes that hospitals implemented under BPCI may have affected patients that are at high-risk for poor outcomes due to clinical risk factors.

It Is important to understand how high-risk patients fared in medical condition episodes. Patients may be excluded from bundled payment-driven care redesign because of clinical risk factors such as frailty or advanced age. Conversely, bundled payment participants may seek greater opportunities for financial savings by targeting high-risk groups as those with larger care process improvement opportunities. In the absence of exclusion or targeting, indiscriminate use of certain strategies across all patients (e.g., reducing SNF length of stay) may not meet the needs of high-risk patients and could also create harm (e.g., increased readmissions).

Despite these potential dynamics, unfortunately little is known about how high-risk patients were affected by BPCI-driven practice redesign for medical condition episodes. Only one study has evaluated clinically high-risk patients in the program, finding that it did not appear to adversely affect outcomes among individuals with dementia and prior institutional care [8]. However, while somewhat reassuring, that study did not compare how high-risk and non-risk patients fared with respect to each other, i.e., whether care patterns or outcomes changed differentially for high-risk and non-high-risk patients under bundled payments.

Evaluating these relationships is critical for understanding the practice and policy benefits of bundled payments—particularly amid other ongoing programs in the US and beyond [9]. Therefore, to address these knowledge gaps, we evaluated changes in care patterns and outcomes for high-risk versus non-high risk patients admitted to BPCI participant hospitals for medical condition episodes.

## 2. Methods

### 2.1. Data & Study Sample

Publicly available data from the Centers for Medicare and Medicaid Services (CMS) were used to identify hospital enrollment in BPCI Model 2. 100% 2011–2016 Medicare claims data were used for patients hospitalized at hospitals participating in BPCI Model 2 for one of four medical condition episodes: chronic obstructive pulmonary disease (COPD), pneumonia, acute myocardial infarction (AMI), and congestive heart failure (CHF). A 20% 2011–2016 random national sample of Medicare claims data was used for patients hospitalized at non-participant hospitals.

Hospital characteristics were obtained from American Hospital Association Annual Survey data, while market and additional hospital characteristics were obtained through data from the Medicare Provider of Service, Beneficiary Summary, and Accountable Care Organization (ACO) files. The Medicare Provider Enrollment, Chain, and Ownership System file was used to identify BPCI episodes from physician group practice participants. Markets were defined using hospital referral regions drawn from the Dartmouth Atlas.

The study sample Ided Medicare fee-for-service beneficiaries admitted to hospitals for any of the four episodes of interest. Episode types were identified based on Medicare Severity-Diagnosis Related Group (MS-DRG) codes as set forth in BPCI program rules: 280–282 for AMI; 291–293 for CHF; 190–192, 202, and 203 for COPD; and 177–179 and 193–195 for pneumonia. Beneficiaries with end stage renal disease or insurance coverage through Medicare Advantage were excluded, as were beneficiaries who died during the index hospital admission, or lacked continuous primary Medicare fee-for-service coverage either during the episode or in the 12 months preceding the beginning of it.

### 2.2. Study Period

The study period ranged from 1 January 2011 through 31 December 2016. This period was separated into a *pre-bundled payment period* (January 2011–September 2013) prior to the start of BPCI and a *bundled payment period* (October 2013–December 2016). 

### 2.3. Hospitals

Hospitals participating in BPCI Model 2 during the study period for any of the four episodes of interest were defined as *BPCI hospitals*. This designation was time-varying, reflecting hospital-condition level entry into or exit from BPCI over time. Once hospitals began BPCI participation for a given episode type, all subsequent episodes considered to be in BPCI hospital group regardless of any subsequent program dropout. 

*Non-BPCI hospitals* were defined as those that (a) never participated in the program across the study and (b) were also located in markets—as defined by hospital referral regions—with no BPCI hospitals across the study period [10]. This approach for defining non-BPCI hospitals was used in order to minimize potential bias arising from BPCI entry and exit over time and unobservable changes at non-BPCI hospitals as a result of “spillover” effects from BPCI hospitals (changes in characteristics of patients receiving care at non-BPCI hospitals due to changes at BPCI hospitals, but that were unobservable from study data). Non-BPCI hospitals with fewer than 10 admissions in the BPCI period for the included medical condition episodes were also excluded.

### 2.4. Episode Construction

Following prior work and BPCI Model 2 rules, 90-day episodes were constructed beginning with hospital admission and spanned 90 days after hospital discharge. To avoid bias arising from Medicare rules related to precedence—rules for handling how overlapping episodes are assigned to hospitals—this analysis also followed prior methods and constructed naturally occurring episodes by assigning overlapping ones to the earlier hospital admission [7,11]. All episodes associated with physician group practice, rather than hospital, BPCI Model 2 participants were removed.

### 2.5. High-Risk Patient Groups

Five high-risk patient groups were used in this analysis: advanced age (patients over the age of 85 years), high case-mix (defined as individuals in the top quintile of Elixhauser mortality scores among patients nationwide), disabled, frail (based on diagnoses from claims data), and prior institutional post-acute care provider utilization (inpatient rehabilitation facilities [IRFs] and SNFs) [12,13].

### 2.6. Variables

Study exposures corresponded to the five high-risk patient groups. In particular, for each group, the exposure was the interaction between membership in that high-risk group and hospitalization at a BPCI hospital. The analysis used a time-varying indicator of BPCI participation by defining it as 1 for episodes occurring during the BPCI period at BPCI hospitals after they started participation, and 0 otherwise. This design reflected the time-varying nature of BPCI participation.

There were two pre-specified primary outcomes: SNF length of stay measured in days, meant to reflect the care redesign driven by BPCI Model 2 for medical condition episodes1; and 90-day unplanned readmission rate, meant to capture potential unintended harms of SNF length of stay reductions. Secondary outcomes included 90-day post-discharge mortality rate, 90-day post-discharge spending standardized and adjusted to 2016 dollars, and several measures of post-acute care utilization: discharge to institutional post-acute care providers (SNFs or IRFs), discharge home with home health agency (HHA) services). 

Based on prior studies, covariates were included at the patient-level, such as age, sex, Elixhauser comorbidities, and market-level, such as population size and Medicare Advantage penetration [14,15,16,17,18].

### 2.7. Statistical Analysis

Following prior studies, for each of the four episode types, propensity scores with replacement were used to match BPCI hospitals and non-BPCI hospitals using hospital and market characteristics from 2011 (Supplementary Methods) [16,19]. Each BPCI hospital was allowed to be matched with up to 3 non-BPCI hospitals, within a caliper of 0.2 of the standard deviation of the log-odds propensity score [20]. Characteristics of propensity-matched BPCI and non-BPCI hospitals were compared using standardized differences of means and proportions [21].

Adjusted analyses were conducted using a series of difference-in-differences (DID) generalized linear models with identity links and normal distributions to evaluate the relationship between BPCI participation and medical condition episode outcomes. An interaction term between an indicator of membership in each high-risk group and the time-varying BPCI participation term was used to examine differential changes in outcomes for high-risk versus non-high-risk patients. All models included hospital, MS-DRG, and time (quarter-year) fixed effects and controlled for patient and time-varying hospital and market characteristics, with standard errors clustered at the hospital level. These variables and models were applied to data from the baseline period to examine consistency with the parallel trends assumption. Wald tests did not indicate divergent baseline period trends in outcomes (Table S1).

We tested the robustness of main analysis findings. via a “stayers only” sensitivity analysis in which we compared outcomes between BPCI hospitals that stayed in the program through the end of the study period (2016 Quarter 3) and propensity-matched non-BPCI hospitals. Statistical tests were two-tailed and considered significant at alpha = 0.05 for the primary outcome. All analyses were conducted using SAS (version 9.4, SAS Institute, Inc., Cary, NC, USA). 

The University of PennsylIania Institutional Review Board approved this study with a waiver of informed consent. 

## 3. Results

The study sample consisted of 471,421 patients hospitalized at 226 BPCI and 700 propensity-matched non-BPCI Hospitals. BPCI and non-BPCI hospitals differed with respect to in hospital, market, and episode characteristics at the start of the pre-bundled payment period (Table 1 and Table S2). Compared to non-BPCI hospitals, BPCI hospitals tended to be larger, non-profit, teaching hospitals located in urban areas and markets with larger populations and smaller proportions of low-income individuals. 

Differences between hospital groups decreased after propensity score matching, with standardized mean differences less than or equal to 0.2 for all variables with the exception of geographic distribution, which had a post-match standardized difference of 0.6 and was not included in the propensity matching model (Supplementary Methods). Medicare Advantage and accountable care organization penetration tended to be higher in markets with BPCI hospitals than markets without any BPCI hospitals.

Some patient characteristics differed between BPCI versus non-BPCI hospitals (Table 2). Compared to those admitted to non-BPCI hospitals, more patients admitted to BPCI hospitals had advanced age and prior IRF/SNF utilization and fewer were disabled. In contrast, the average age, and gender distribution were similar for patients admitted to BPCI versus non-BPCI hospitals. The proportion of patients in other high-risk groups were comparable between the two hospital groups. 

### 3.1. Primary Outcomes

**Figure 1:**
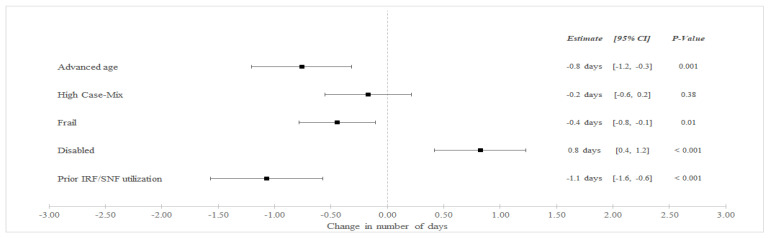
Adjusted Changes in in SNF LOS for Medical Condition Episodes Among High-Risk Patients (2011–2016). Abbreviations: LOS = length of stay; IRF = Inpatient Rehabilitation Facility; SNF = Skilled Nursing Facility. Advanced age = Age > 85 years old. High Case-Mix = Top 20% of Elixhauser score. Prior IRF/SNF Utilization = Utilization within the preceding 12 months.

In unadjusted analyses (Table 3), SNF LOS decreased among BPCI hospitals from an average of 8.9 days in the pre-bundled payment period to 8.4 in the bundled payment but increased among non-BPCI Hospitals from an average of 7.6 to 7.8 days between the pre-bundled payment and bundled payment periods, respectively. In adjusted difference-in-differences (aDID) analysis (Figure 1), BPCI participation was associated with a differentially lower in SNF LOS among frail patients (aDID −0.4 days versus non-frail patients, 95% CI −0.8 to −0.1 days, *p* = 0.01), patients with advanced age (aDID −0.8 days versus younger patients, 95% CI −1.2 to −0.3 days, *p* = 0.001), and patients with prior SNF/IRF utilization (aDID −1.1 days versus patients without prior utilization, 95% CI −1.6 to −0.6 days, *p* < 0.001). BPCI participation was also associated with differentially greater SNF LOS among disabled patients (aDID 0.8 days versus non-disabled patients, 95% CI 0.4 to 1.2 days, *p* < 0.001).

**Figure 2:**
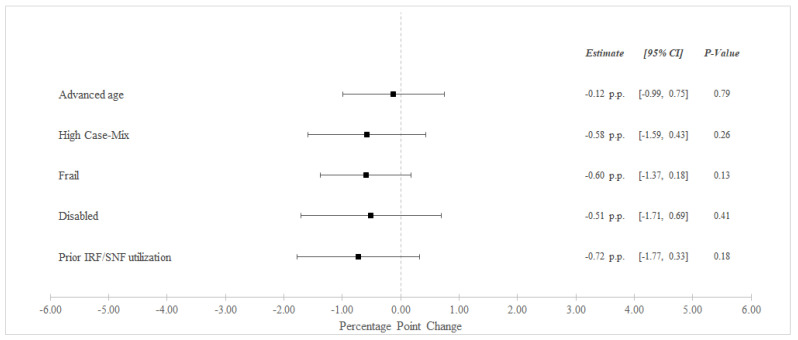
Adjusted Changes in 90-Day Unplanned Readmission Rate ^‡^ for Medical Condition Episodes Among High-Risk Patients (2011–2016). Abbreviations: IRF = Inpatient Rehabilitation Facility; SNF = Skilled Nursing Facility. ^‡^ At least one acute care readmission during the 90-day episode. Advanced age = Age > 85 years old. High Case-Mix = Top 20% of Elixhauser score. Prior IRF/SNF Utilization = Utilization within the preceding 12 months.

Between the pre-bundled payment and bundled payment periods, unadjusted 90-day readmissions decreased from 31.8% to 30.7% among BPCI Hospitals and from 32.5% to 31.9% among non-BPCI Hospitals (Table 3). In adjusted analysis, BPCI participation was not associated with differential changes in 90-day readmissions for any high-risk versus non-high risk groups (Figure 2).

### 3.2. Secondary Outcomes

In unadjusted analyses, there were changes in secondary outcomes between the pre-bundled payment and bundled payment periods (Table S3). Differential changes were also observed for some outcomes in adjusted analyses (Figures S1–S4). In particular, BPCI participation was also associated with differentially lower post-discharge spending for frail patients (aDID −$401 versus non-frail patients, 95% CI −$682 to −$120, *p* = 0.01) and patients with prior SNF/IRF utilization (aDID −$534 versus patients without prior utilization, 95% CI −$922 to −$145, *p* = 0.01). BPCI participation was associated with differentially lower mortality for disabled patients (aDID −0.66 percentage points [pp] versus non-disabled patients, 95% CI −1.27 to −0.06 pp, *p* = 0.03) and differentially greater discharge to SNF/IRF for frail patients (aDID 1.05 pp versus non-frail patients, 95% CI 0.28 to 1.82 pp, *p* = 0.01).

### 3.3. Sensitivity Analyses

Results from sensitivity analyses using hospitals with stayed through the end of the study period (Figures S5–S10) were qualitatively similar overall to results from the main analyses.

## 4. Discussion

To our knowledge, this was the first analysis to directly evaluate outcomes for high-risk versus other patients under bundled payments. It demonstrated that hospital participation in bundled payments was associated with differentially greater changes in care patterns and certain outcomes for some clinically high-risk patient groups, including reduced SNF LOS, spending and mortality without increases in unplanned readmissions. This study poses several implications.

First, the analysis provides evidence that some changes of medical condition bundles disproportionately applied to high-risk patients. In particular, both SNF LOS and unplanned readmissions decreased to a greater degree at BPCI versus non-BPCI hospitals among several patient groups, i.e., the gaps in both outcomes narrowed for these patients under bundled payments. While these findings were not uniformly observed across groups, they suggest BPCI participants may have focused on, rather than excluded, some high-risk individuals from bundled payment-driven care redesign.

Second, results from this study offer some reassurance against potentially indiscriminate use of strategies in medical bundles and harms to high-risk individuals. For instance, though SNF length of stay decreased overall for individuals receiving medical condition episode care through BPCI [7], length of stay was differentially greater for disabled patients—perhaps reflecting that they were protected from quicker, and potentially premature, discharges. Though a well-known strategy in bundled payments has been shifting discharge location away from SNFs or IRFs toward home, this analysis demonstrated that discharge to these institutional PAC providers was differentially greater among frail patients. No high-risk groups exhibited differentially greater readmissions. Though these findings should be further investigated in future work, they are nonetheless reassuring when taken together as policy and practice leaders continue to implement medical bundles.

Third, study findings underscore the need for additional work evaluating how bundled payments and other value-based payment models affect equity among different vulnerable groups. In particular, future studies could build on insights from the variation observed in this analysis and assess how other payment models influence patient access and quality outcomes for clinically high-risk individuals. Such evaluations should be conducted for all payment models to ensure that payment reforms are tool for reducing rather than perpetuating health care disparities [22].

This analysis has limitations. First, as with all observational analyses, findings are subject to residual confounding. However, the analysis utilized a quasi-experimental methodology that addressed these risks by directly accounting for multiple patient, hospital, and market characteristics and also hospital and quarter-year fixed effects. Second, this analysis evaluated one model within one bundled payment program. However, BPCI Model 2 is a critical model to understand as the direct basis for all subsequent CMS bundled payment programs. Third, while this analysis evaluated multiple aspects of patients’ clinical complexity, individuals may be “high risk” due to a number of social or other clinical determinants. Future work should evaluate different high-risk features under voluntary bundled payments.

Nonetheless, this study suggests that bundled payment strategies were not applied indiscriminately to all patients and were instead associated with differential changes in the care of some high-risk groups. Together, these findings offer insight into how high these groups were affected by medical bundles, help allay concerns about harm, and underscores the need to conduct similar studies for other value-based payment models.

## Figures and Tables

**Table 1:** Characteristics of BPCI and Non-BPCI Hospitals for Medical Condition Episodes, Before and After Propensity Score Matching (2011).

	Before Matching			After Matching		
	BPCI Hospitals	Non-BPCI Hospitals	SMD	BPCI Hospitals	Non-BPCI Hospitals	SMD
**Hospitals, No.**	238	1415	N/A	226	700	N/A
**Total Beneficiaries, No.**	475,441	484,335	N/A	268,177	205,397	N/A
**Total Episodes, No.**	618,962	672,497	N/A	336,965	267,521	N/A
**Beneficiaries, No.**						
Acute Myocardial Infarction	27,158	62,616	N/A	25,248	20,673	N/A
Chronic Obstructive Pulmonary Disease	72,220	144,813	N/A	62,315	51,846	N/A
Pneumonia	103,580	193,149	N/A	96,636	72,482	N/A
Congestive Heart Failure	113,462	147,042	N/A	100,603	75,793	N/A
**Hospitals, No.**						
Acute Myocardial Infarction	96	1148	N/A	89	257	N/A
Chronic Obstructive Pulmonary Disease	136	1398	N/A	120	341	N/A
Pneumonia	144	1411	N/A	134	380	N/A
Congestive Heart Failure	185	1386	N/A	168	474	N/A
**Episodes, No.**						
Acute Myocardial Infarction	28,421	66,264	N/A	26,449	21,858	N/A
Chronic Obstructive Pulmonary Disease	93,503	197,625	N/A	80,437	68,670	N/A
Pneumonia	114,746	221,188	N/A	107,065	81,440	N/A
Congestive Heart Failure	138,523	187,420	N/A	123,014	95,553	N/A
**Hospital characteristics**						
**Ownership status, No. (%)**						
For profit	119 (21.3)	1072 (20.3)	0.5	94 (18.3)	256 (17.5)	<0.001
Government	15 (2.7)	927 (17.5)		15 (2.9)	50 (3.4)	
Not-for-profit	424 (76.0)	3295 (62.2)		405 (78.8)	1154 (79.0)	
**Urban, No. (%)**	520 (93.2)	3433 (64.9)	0.7	476 (92.6)	1335 (91.4)	0.04
**Geographic Location, No. (%) ^†^**						
Midwest	41 (17.2)	402 (28.4)		41 (18.1)	222 (31.7)	
Northeast	77 (32.3)	103 (7.3)	0.7	74 (32.7)	69 (9.9)	0.6
South	79 (33.2)	618 (43.7)		74 (32.7)	230 (32.9)	
West	41 (17.2)	292 (20.6)		37 (16.4)	179 (25.9)	
Teaching status, No. (%) *						
Major teaching	59 (10.6)	354 (6.7)	0.3	58 (11.3)	155 (10.6)	0.02
Minor teaching	225 (40.3)	1541 (29.1)		199 (38.7)	559 (38.3)	
Non-teaching	274 (49.1)	3399 (64.2)		257 (50.0)	746 (51.1)	
Ratio of medical and dental residents to total beds, mean (SD) **	8.5 (19.7)	4.9 (16.1)	0.2	8.7 (19.8)	8.2 (20.9)	0.03
**Disproportionate share, mean $ (SD)** **^‡^**	5,436,588 (6,898,391)	3,087,284 (4,802,784)	0.4	5,303,249 (6,836,720)	4,642,904 (6,359,659)	0.1
**Medicare days as % of total patient days, mean % (SD)**	27.6 (7.4)	24.8 (9.0)	0.3	27.4 (7.5)	26.9 (7.9)	0.06
**Total hospital beds, mean (SD)**	321 (252)	205 (179)	0.5	316 (238)	292 (220)	0.1
**Hospital market characteristics ^±^**						
**Population, mean (SD)**	2,211,711 (1,925,970)	1,145,138 (900,691)	0.7	1,840,333 (1,386,232)	1,627,170 (1,078,766)	0.17
**Low-income status, mean % (SD)**	18.6 (13.7)	27.0 (18.9)	−0.5	18.9 (14.1)	19.6 (15.6)	−0.04
**Medicare Advantage penetration, mean % (SD)**	25.7 (11.7)	24.1 (14.2)	0.1	25.6 (11.8)	25.5 (14.5)	−0.01
**SNF beds per 10,000 patients, mean (SD)**	10,665 (9448)	6320 (4777)	0.6	9178 (7917)	7986 (5297)	0.2
**Hospital market share, mean % (SD)**	40.6 (43.8)	36.9 (46.2)	0.1	43.1 (44.5)	43.7 (47.8)	−0.01
**Hospital HHI, mean (SD)** **^ǂ^**	1587.8 (1423.2)	2099.9 (1542.4)	−0.3	1690.3 (1440.5)	1773.8 (1600.9)	−0.05
**Hospital utilization characteristics**						
**BPCI-related hospital discharges, mean % (SD)** **^ǂǂǂ^**	34.8 (6.2)	37.4 (8.2)	−0.4	34.8 (6.3)	35.3 (6.9)	−0.07
**Proportion of discharges to highest-volume SNF, mean % (SD)**	27.8 (15.2)	38.0 (22.4)	−0.5	27.9 (14.8)	29.4 (17.3)	−0.1
**Proportion of discharges to highest-volume IRF, mean % (SD)**	56.9 (43.4)	47.1 (46.9)	0.2	56.4 (43.6)	54.2 (46.2)	0.05

Notes: BPCI = Bundled Payments for Care Improvement initiative; HHI = Herfindahl-Hirschman Index; IRF = Inpatient Rehabilitation Facility; SD = Standard Deviation; SNF = Skilled Nursing Facility. * Major teaching is a hospital with a Council of Teaching Hospitals designation. Minor teaching refers to non-Council hospitals with approved residency training programs. ** Measure of the size of the teaching program used in Medicare’s teaching adjustment. Means are shown because the standardized differences were calculated with means. **^†^** Not used in propensity-score matching. **^‡^** Amount paid to the hospital under the disproportionate share program reflecting the indigent population served. ^±^ Market is defined by Hospital Referral Region. ^ǂ^ Measure of hospital market concentration. ^ǂǂǂ^ Proportion of annual admissions for 10 highest-volume BPCI conditions (by total hospital and physician group episodes). Hospital, beneficiary, and episode counts are shown, along with characteristics used for propensity score matching (except geographic distribution added during review process). Hospital and episode counts are total counts for the study period (2011–2016). Hospital, hospital market, and hospital utilization characteristics used 2011 as the first year of the study period, which was used to match hospitals. BPCI hospitals were matched with Non-BPCI hospitals in markets without BPCI hospital participants (Non-BPCI markets). BPCI hospitals were propensity-score matched by condition with up to three Non-BPCI hospitals, using 0.2 of the log odds propensity score.

**Table 2:** Characteristics of Patients Hospitalized for Medical Condition Episodes to BPCI and Non-BPCI Hospitals (2011–2016).

	BPCI Hospitals		Non-BPCI Hospitals		
	Baseline Period	Intervention Period	Baseline Period	Intervention Period	DiD *
**Hospitals, No.**	226	226	699	699	N/A
**Beneficiaries, No.**	148,900	138,666	113,900	108,938	N/A
**Total episodes, No.**	175,261	161,704	137,126	130,395	N/A
**Episodes, No.**					
Acute Myocardial Infarction	13,356	13,093	10,874	10,688	N/A
Chronic Obstructive Pulmonary Disease	44,157	35,843	39,017	33,006	N/A
Pneumonia	58,406	49,470	45,709	40,709	N/A
Congestive Heart Failure	62,461	66,092	48,565	51,632	N/A
**Patient characteristics**					
**Age, mean age (SD)**	77.1 (12.3)	77.3 (12.2)	75.6 (12.6)	75.9 (12.5)	−0.1
**Female, No. (%)**	97,884 (55.9)	89,788 (55.5)	76,160 (55.5)	71,689 (55.0)	−3625
**Elixhauser score, mean score (SD)**	19.3 (13.8)	19.4 (13.8)	19.3 (13.8)	19.5 (13.9)	−0.1
**High Risk Groups**					
**Advanced age, No. (%) ^a^**	48,752 (27.8)	46,217 (28.6)	32,309 (23.6)	32,228 (24.7)	−2454
**High Case-Mix, No. (%) ^b^**	38,078 (21.7)	31,592 (19.5)	30,107 (22.0)	25,415 (19.5)	−1794
**Frail, No. (%)**	67,762 (38.7)	65,879 (40.7)	50,409 (36.8)	51,796 (39.7)	−3270
**Disabled, No. (%)**	22,526 (12.9)	20,074 (12.4)	21,331 (15.6)	19,490 (14.9)	−611
**Prior IRF/SNF utilization, No. (%) ^c^**	33,723 (19.2)	31,011 (19.2)	24,501 (17.9)	24,145 (18.5)	−2356

Notes: BPCI = Bundled Payments for Care Improvement initiative; DiD = Difference-in-differences; IRF = Inpatient Rehabilitation Facility; SNF = Skilled Nursing Facility; SD = Standard Deviation. * Difference-in-differences for categorical outcomes are shown as percentage points. The baseline period spanned 1 January 2011–30 September 2013. The treatment period varied by hospital-condition based on the date of entry so as to maintain consistency with the analytic models, with the earliest start possible being 1 October 2013 when BPCI began. ^a^ Age > 85 years old. ^b^ Top 20% of Elixhauser score. ^c^ Within preceding 12 months.

**Table 3:** Unadjusted Primary Outcomes for Medical Condition Episodes among High-Risk Patients (2011–2016).

	BPCI Hospitals		Non-BPCI Hospitals			
	Baseline Period	Intervention Period	Baseline Period	Intervention Period	DiD *	Percent Change
**SNF LOS, mean days (SD)**	8.9 (19.8)	8.4 (18.7)	7.6 (18.5)	7.8 (18.3)	−0.6	−7.3
Advanced age ^a^	13.7 (23.0)	12.5 (21.3)	12.6 (22.3)	12.2 (21.4)	−0.7	−5.0
High Case-Mix ^b^	11.1 (21.5)	10.3 (20.0)	9.9 (20.6)	9.9 (20.1)	−0.8	−7.5
Frail	13.9 (23.8)	13.0 (22.3)	12.4 (22.9)	12.3 (22.2)	−0.8	−5.9
Disabled	3.6 (13.5)	3.9 (13.8)	3.0 (12.5)	3.3 (12.6)	0.1	1.5
Prior IRF/SNF utilization ^c^	17.9 (25.8)	16.4 (24.0)	16.6 (25.0)	15.9 (24.1)	−0.7	−4.2
**90-day unplanned readmission rate, No. (%) ^‡^**	55,753 (31.8)	49,627 (30.7)	44,622 (32.5)	41,601 (31.9)	−0.5	−1.5
Advanced age ^a^	14,923 (30.6)	13,426 (29.0)	9627 (29.8)	9350 (29.0)	−0.8	−2.5
High Case-Mix ^b^	14,967 (39.3)	11,568 (36.6)	12,161 (40.4)	9511 (37.4)	0.3	0.7
Frail	25,374 (37.4)	23,582 (35.8)	19,524 (38.7)	19,392 (37.4)	−0.4	−1.0
Disabled	7887 (35.0)	6809 (33.9)	7787 (36.5)	7127 (36.6)	−1.2	−3.3
Prior IRF/SNF utilization ^c^	13,535 (40.1)	11,980 (38.6)	10,167 (41.5)	9813 (40.6)	−0.7	−1.6

Notes: BPCI = Bundled Payments for Care Improvement initiative; DiD = Difference-in-differences; LOS = length of stay; SD = Standard Deviation; IRF = Inpatient Rehabilitation Facility; SNF = Skilled Nursing Facility. * Difference-in-differences for categorical outcomes are shown as percentage points. **^‡^** At least one acute care readmission during the 90-day episode. The baseline period spanned 1 January 2011–30 September 2013. The treatment period varied by hospital-condition based on the date of entry so as to maintain consistency with the analytic models, with the earliest start possible being 1 October 2013 when BPCI began. ^a^ Age > 85 years old. ^b^ Top 20% of Elixhauser score. ^c^ Within preceding 12 months.

## Data Availability

Data presented in this study are unable to be shared because of identifiability and cell size limitations by The Centers for Medicare & Medicaid Services.

## Supplementary Materials

The following supporting information can be downloaded at: https://www.mdpi.com/article/10.3390/healthcare10122510/s1, Supplementary Methods. Propensity Score Matching. Table S1. Examination of Parallel Trends Assumption in the Pre-Bundled Payment Period. Table S2. Characteristics of Markets with and without BPCI Hospitals (2011–2016). Table S3. Unadjusted Secondary Outcomes for Medical Condition Episodes among High-Risk Patients (2011–2016). Figure S1. Adjusted CIanges in Post-Discharge Spending for Medical Condition Episodes Among High-Risk Patients (2011–2016). Figure S2. Adjusted Changes in 90-Day Mortality Rate† for Medical Condition Episodes Among High-Risk Patients (2011–2016). Figure S3. Adjusted Changes in Discharge to Institutional PAC Providers for Medical Condition Episodes Among High-Risk Patients (2011–2016). Figure S4. Adjusted Changes in Discharge Home with HHA for Medical Condition Episodes Among High-Risk Patients (2011–2016). Figure S5. Adjusted Changes in SNF LOS for Medical Condition Episodes Among High-Risk Patients (2011–2016) Among Stayer-Hospitals Only. Figure S6. Adjusted Changes in 90-Day Unplanned Readmission Rate‡ for Medical Condition Episodes Among High-Risk Patients (2011–2016) Among Stayer-Hospitals Only. Figure S7. Adjusted Changes in Post-Discharge Spending for Medical Condition Episodes Among High-Risk Patients (2011–2016) Among Stayer-Hospitals Only. Figure S8. Adjusted Changes in 90-Day Mortality Rate† for Medical Condition Episodes Among High-Risk Patients (2011–2016) Among Stayer-Hospitals Only. Figure S9. Adjusted Changes in Discharge to Institutional PAC Providers for Medical Condition Episodes Among High-Risk Patients (2011–2016) Among Stayer-Hospitals Only. Figure S10. Adjusted Changes in Discharge Home with HHA for Medical Condition Episodes Among High-Risk Patients (2011–2016) Among Stayer-Hospitals Only. Reference [23] is cited in the supplementary materials.
