# Supplementary material for: The Association between Bundled Payment Participation and Changes in Medical Episode Outcomes among High-Risk Patients
Source: Healthcare (Basel). 2022 Dec 12;10(12):2510. doi: 10.3390/healthcare10122510 (PMC9778756; doi:10.3390/healthcare10122510)
Supplement: Supplementary file 1 [file healthcare-10-02510-s001.zip › healthcare-2011187-supplementary.pdf]

Supplement for "The Association between Bundled Payment Participation and Changes in Medical Episode Outcomes among High-Risk Patients"

Contents

Supplementary Methods. Propensity Score Matching

Table S1. Examination of Parallel Trends Assumption in the Pre-Bundled Payment Period

Table S2. Characteristics of Markets with and without BPCI Hospitals (2011-2016)

Table S3. Unadjusted Secondary Outcomes for Medical Condition Episodes among High-Risk Patients (2011-2016)

Figure S1. Adjusted Changes in Post-Discharge Spending for Medical Condition Episodes Among High-Risk Patients (2011-2016)

Figure S2. Adjusted Changes in 90-Day Mortality Rate† for Medical Condition Episodes Among High-Risk Patients (2011-2016)

Figure S3. Adjusted Changes in Discharge to Institutional PAC Providers for Medical Condition Episodes Among High-Risk Patients (2011-2016)

Figure S4. Adjusted Changes in Discharge Home with HHA for Medical Condition Episodes Among High-Risk Patients (2011-2016)

Figure S5. Adjusted Changes in SNF LOS for Medical Condition Episodes Among High-Risk Patients (2011-2016) Among Stayer-Hospitals Only

Figure S6. Adjusted Changes in 90-Day Unplanned Readmission Rate‡ for Medical Condition Episodes Among High-Risk Patients (2011-2016) Among Stayer-Hospitals Only

Figure S7. Adjusted Changes in Post-Discharge Spending for Medical Condition Episodes Among High-Risk Patients (2011-2016) Among Stayer-Hospitals Only

Figure S8. Adjusted Changes in 90-Day Mortality Rate† for Medical Condition Episodes Among High-Risk Patients (2011-2016) Among Stayer-Hospitals Only

Figure S9. Adjusted Changes in Discharge to Institutional PAC Providers for Medical Condition Episodes Among High-Risk Patients (2011-2016) Among Stayer-Hospitals Only

Figure S10. Adjusted Changes in Discharge Home with HHA for Medical Condition Episodes Among High-Risk Patients (2011-2016) Among Stayer-Hospitals Only

Supplementary Methods. Propensity Score Matching

We used propensity score matching to decrease differences between BPCI and non-BPCI hospitals. In particular, we used logistic regression with baseline characteristics to estimate a propensity score (value between 0 and 1) for hospital to be enrolled in BPCI in order to match BPCI and Non-BPCI participant hospitals. We used a greedy 3:1 approach and allowed BPCI hospitals to be matched with 3 non-BPCI hospitals, respectively[1]. This process achieved good overlap between the BPCI and Non-BPCI groups based on the propensity scores, as demonstrated through the common support graphs below.

**CHF**		**AMI**	
Before Matching	After Matching	Before Matching	After Matching
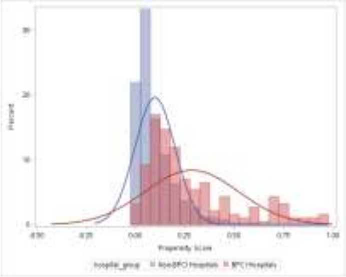	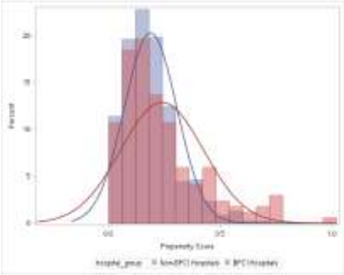	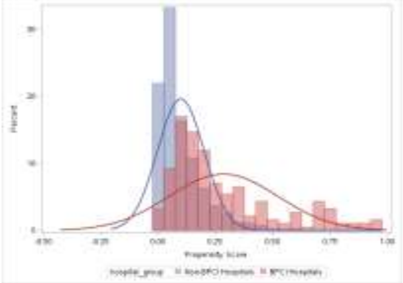	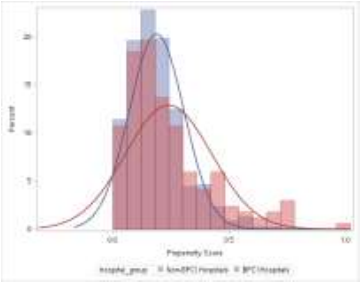
**Pneumonia**		**COPD**	
Before Matching	After Matching	Before Matching	After Matching
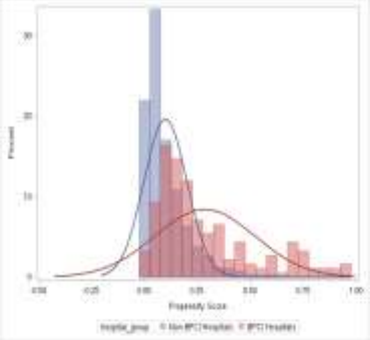	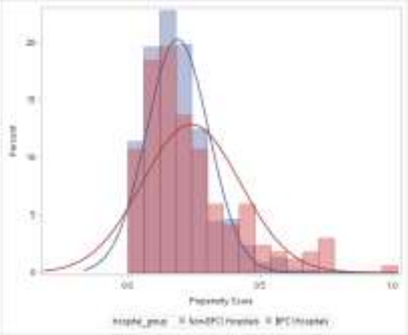	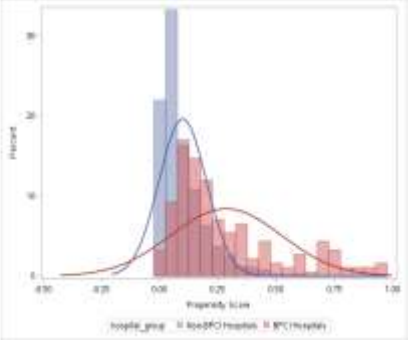	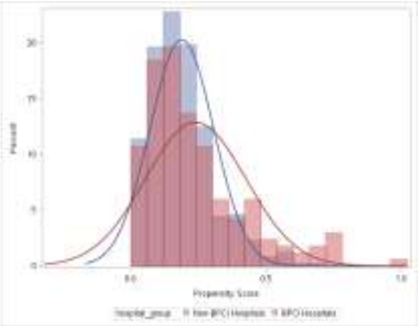

1. Gu XS, Rosenbaum PR. Comparison of Multivariate Matching Methods: Structures, Distances, and Algorithms. *Journal of Computational and Graphical Statistics* 1993;2(4):405-20. doi: 10.1080/10618600.1993.10474623

Table S1. Examination of Parallel Trends Assumption in the Pre-Bundled Payment Period

<table>
<tr><td>SNF LOS</td><td>90-day readmission rate**</td></tr>
<tr><td>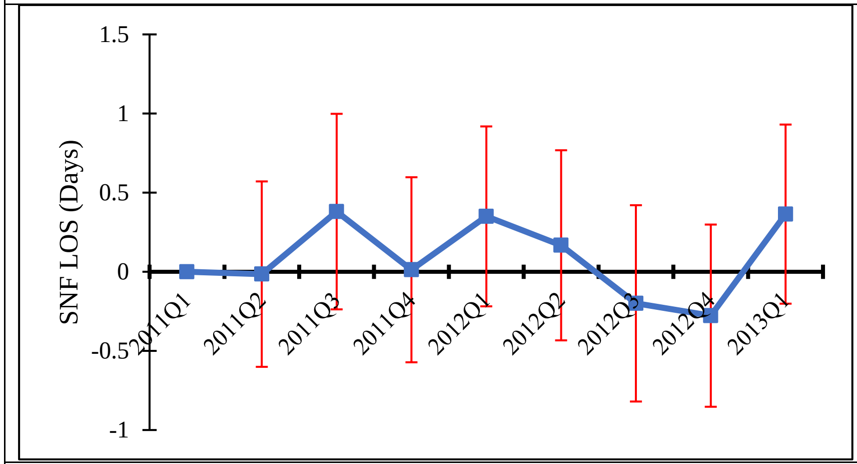
</td><td>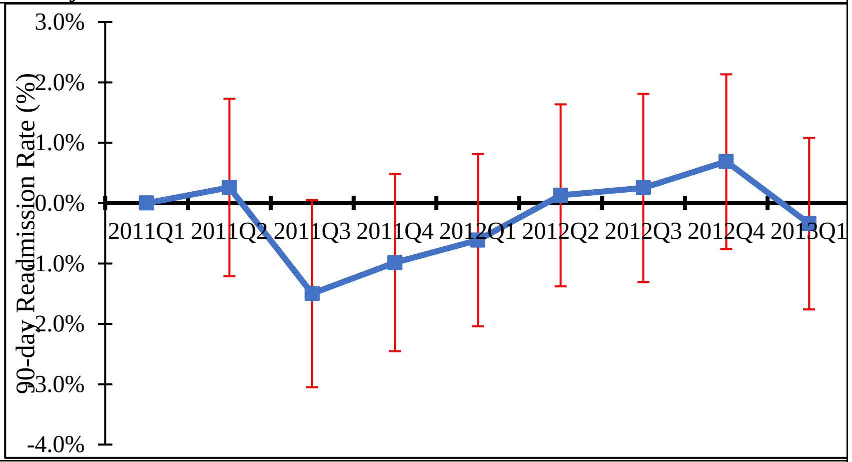
</td></tr>
<tr><td>Post-discharge spending</td><td>90-day mortality rate*</td></tr>
<tr><td>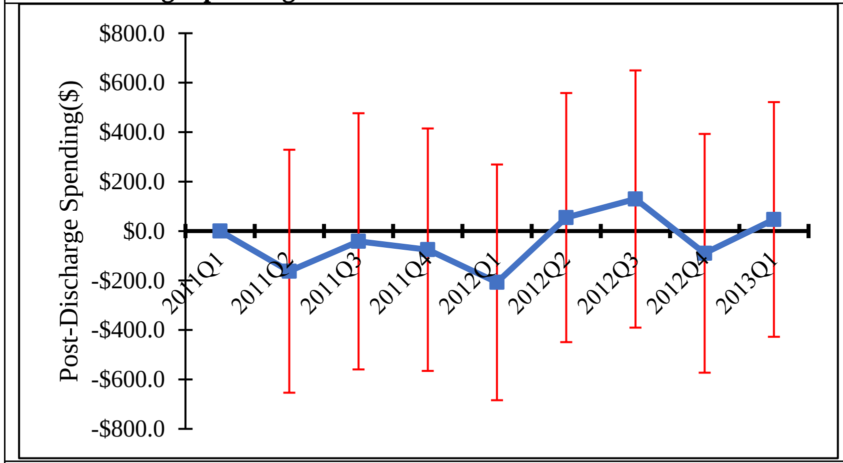
</td><td>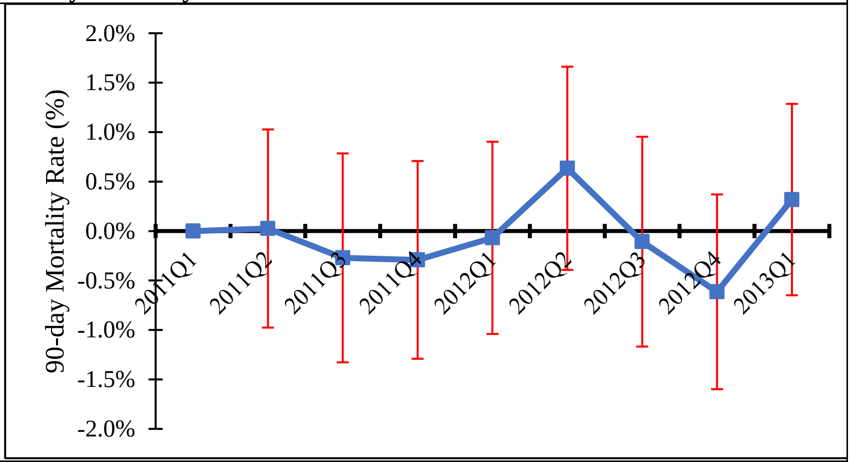
</td></tr>
<tr><td>Discharge to institutional PAC providers</td><td>Discharge home with HHA</td></tr>
</table>

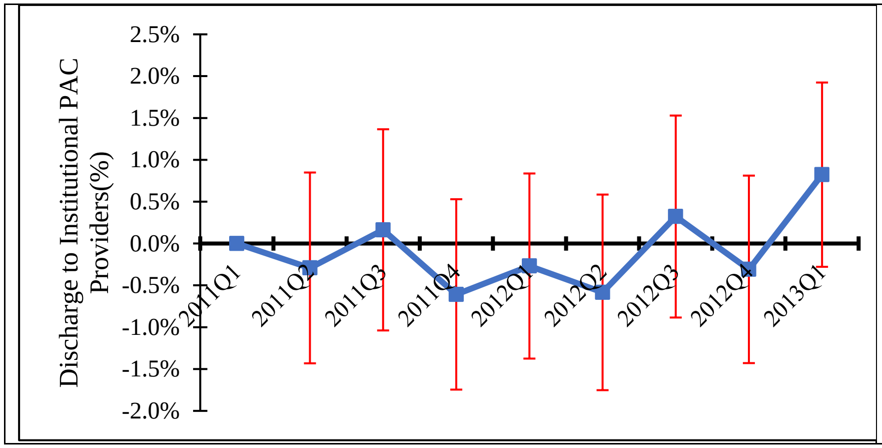

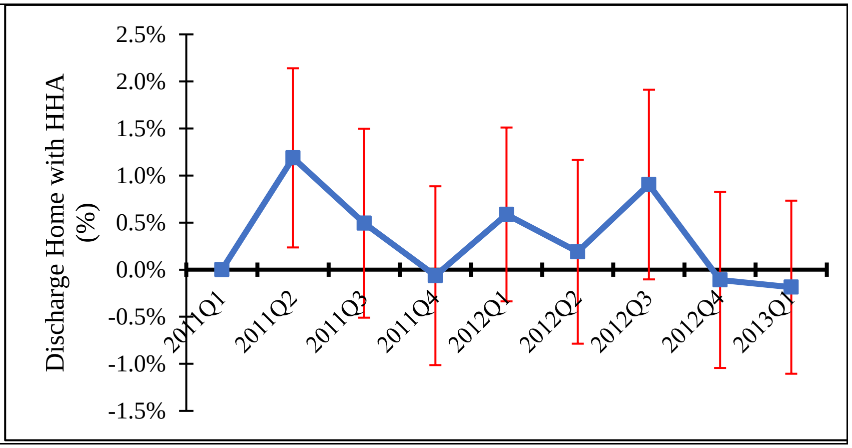

Abbreviations: HHA=Home Health Agency, PAC=Post-acute care. *At least one acute care readmission during the 90-day episode.**Percentage who died within 90 days after discharge alive from index hospitalization. The parallel trends assumption was tested for each outcome by modeling the relationship between time and BPCI versus non-BPCI hospital group status in the pre-bundled payment period (January 1, 2011 - September 30, 2013).

Table S2. Characteristics of Markets with and without BPCI Hospitals (2011-2016)

	Markets with BPCI Hospitals‡		Markets without BPCI Hospitals‡		
	**Baseline Period**	**Intervention Period**	**Baseline Period**	**Intervention Period**	**Unadjusted DiD***
ACO penetration, mean %±	21.2	22.4	15.8	15.8	1.2
MA penetration, mean %ǂ	24.7	29.3	23.5	27.7	0.4
Beneficiary population, mean (SD)	116,078 (97,768)	117,802 (99,446)	63,656 (48,770)	64,775 (50,005)	605

Abbreviations: ACO=Accountable Care Organization; BPCI=Bundled Payments for Care Improvement initiative; DiD=Difference-in-differences; MA=Medicare Advantage; SD=Standard Deviation. ‡Market is defined by Hospital Referral Region. ±Determined from a random 20% sample of fee-for-service beneficiaries and the CMS ACO Provider-level Research Identifiable File. ǂDetermined by the Medicare Beneficiary Summary File and computing at a market–quarter level the proportion of Medicare beneficiaries in Medicare Advantage any time during that quarter. *Difference-in-differences for categorical outcomes are shown as percentage points. The baseline period spanned January 1, 2011 – September 30, 2013. The treatment period varied by hospital-condition based on the date of entry so as to maintain consistency with the analytic models, with the earliest start possible being October 1, 2013 when BPCI began.

Table S3. Unadjusted Secondary Outcomes for Medical Condition Episodes among High-Risk Patients (2011-2016)

	BPCI Hospitals		Non-BPCI Hospitals			
	**Baseline Period**	**Intervention Period**	**Baseline Period**	**Intervention Period**	**DiD***	**Percent Change**
**Post-discharge spending, mean $ (SD)**	18,893 (16,181)	19,346 (16,159)	18,372 (15,812)	19,223 (16,238)	-398	-2.1
Advanced age[a]	20,396 (15,701)	20,559 (15,547)	19,629 (15,415)	20,160 (15,482)	-369	-1.8
High Case-Mix[b]	22,505 (17,390)	22,383 (17,052)	22,069 (17,223)	22,240 (17,120)	-293	-1.3
Frail	22,928 (17,528)	23,034 (17,308)	22,464 (17,295)	23,033 (17,550)	-463	-2.0
Disabled	16,828 (15,379)	17,569 (15,681)	16,893 (15,277)	17,932 (16,160)	-298	-1.8
Prior IRF/SNF utilization[c]	25,256 (18,320)	25,311 (18,207)	24,849 (17,953)	25,390 (18,386)	-486	-1.9
**90-day mortality rate, No. (%)†**	21,959 (12.5)	20,016 (12.4)	15,260 (11.1)	14,980 (11.5)	-0.5	-4.1
Advanced age[a]	10,100 (20.7)	9,174 (19.8)	6,219 (19.2)	6,387 (19.8)	-1.4	-6.9
High Case-Mix[b]	8,213 (21.6)	6,690 (21.2)	5,922 (19.7)	5,092 (20.0)	-0.8	-3.5
Frail	12,456 (18.4)	11,892 (18.1)	8,436 (16.7)	8,615 (16.6)	-0.2	-1.2
Disabled	1,075 (4.8)	923 (4.6)	908 (4.3)	875 (4.5)	-0.4	-8.5
Prior IRF/SNF utilization[c]	6,744 (20.0)	6,010 (19.4)	4,557 (18.6)	4,419 (18.3)	-0.3	-1.6
**Discharge to institutional PAC, No. (%)**	20,528 (15.0)	19,891 (15.3)	31,125 (17.8)	28,420 (17.6)	-0.5	-2.6
Advanced age[a]	8,866 (27.4)	8,679 (26.9)	14,452 (29.6)	13,254 (28.7)	-0.5	-1.5
High Case-Mix[b]	5,996 (19.9)	5,082 (20.0)	8,647 (22.7)	7,075 (22.4)	-0.4	-1.7
Frail	12,026 (23.9)	12,425 (24.0)	18,493 (27.3)	17,693 (26.9)	-0.6	-2.1
Disabled	1,002 (4.7)	986 (5.1)	1,274 (5.7)	1,282 (6.4)	0.4	6.5
Prior IRF/SNF utilization[c]	8,091	7,564	12,076	10,600	0.1	0.2

	(33.0)	(31.3)	(35.8)	(34.2)		
**Discharge home with HHA, No. (%)**	18,977 (10.8)	16,864 (10.4)	11,656 (8.5)	10,313 (7.9)	0.2	1.8
Advanced age[a]	6,525 (13.4)	5,934 (12.8)	3,617 (11.2)	3,247 (10.1)	0.6	4.3
High Case-Mix[b]	4,495 (11.8)	3,498 (11.1)	2,898 (9.6)	2,225 (8.8)	0.1	1.2
Frail	7,240 (10.7)	6,620 (10.0)	4,430 (8.8)	4,251 (8.2)	-0.1	-0.5
Disabled	1,302 (5.8)	1,192 (5.9)	978 (4.6)	847 (4.3)	0.4	6.9
Prior IRF/SNF utilization[c]	2,795 (8.3)	2,513 (8.1)	1,723 (7.0)	1,623 (6.7)	0.1	1.5

Abbreviations: BPCI=Bundled Payments for Care Improvement initiative; DiD=Difference-in-differences; HHA=Home Health Agency; LOS=length of stay; PAC=Post-acute Care; SD=Standard Deviation; IRF=Inpatient Rehabilitation Facility; SNF=Skilled Nursing Facility. [*]Difference-in-differences for categorical outcomes are shown as percentage points. [†]Percentage who died within 90 days after discharge alive from index hospitalization. [‡]At least one acute care readmission during the 90-day episode. The baseline period spanned January 1, 2011 – September 30, 2013. The treatment period varied by hospital-condition based on the date of entry so as to maintain consistency with the analytic models, with the earliest start possible being October 1, 2013 when BPCI began. [a]Age >85 years old. [b]Top 20% of elixhauser score. [c]Within preceding 12 months.

Figure S1. Adjusted Changes in Post-Discharge Spending for Medical Condition Episodes Among High-Risk Patients (2011-2016)

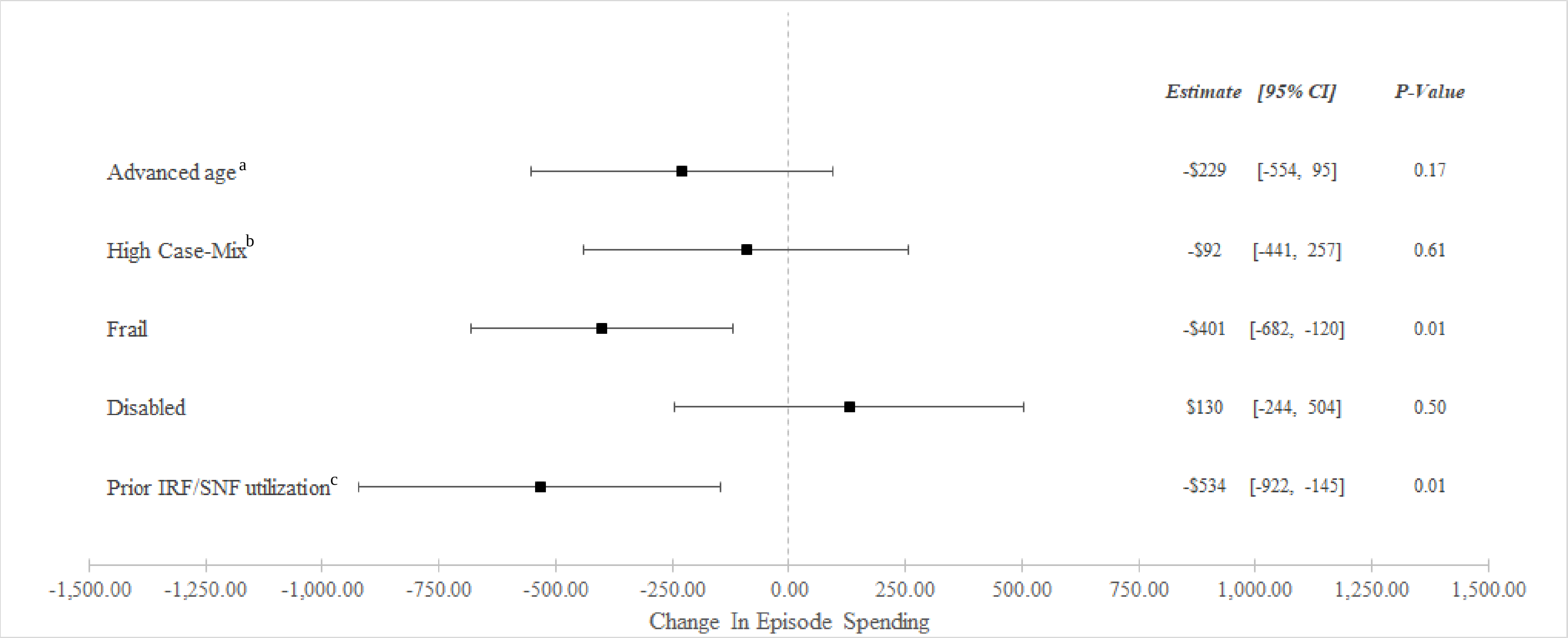

Abbreviations: IRF=Inpatient Rehabilitation Facility; SNF=Skilled Nursing Facility. [a]Age >85 years old. [b]Top 20% of elixhauser score. [c]Within preceding 12 months.

Figure S2. Adjusted Changes in 90-Day Mortality Rate† for Medical Condition Episodes Among High-Risk Patients (2011-2016)

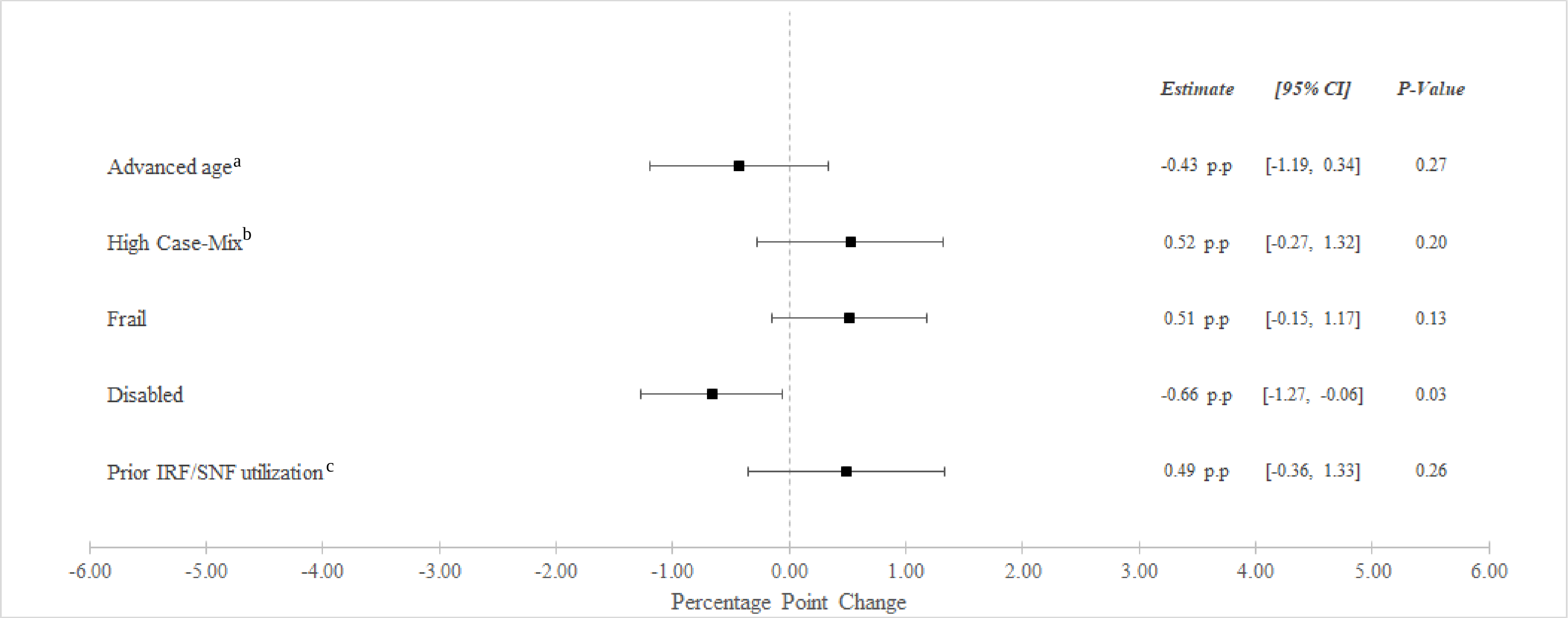

Abbreviations: IRF=Inpatient Rehabilitation Facility; SNF=Skilled Nursing Facility. †Percentage who died within 90 days after discharge alive from index hospitalization. [a]Age >85 years old. [b]Top 20% of elixhauser score. [c]Within preceding 12 months.

Figure S3. Adjusted Changes in Discharge to Institutional PAC Providers for Medical Condition Episodes Among High-Risk Patients (2011-2016)

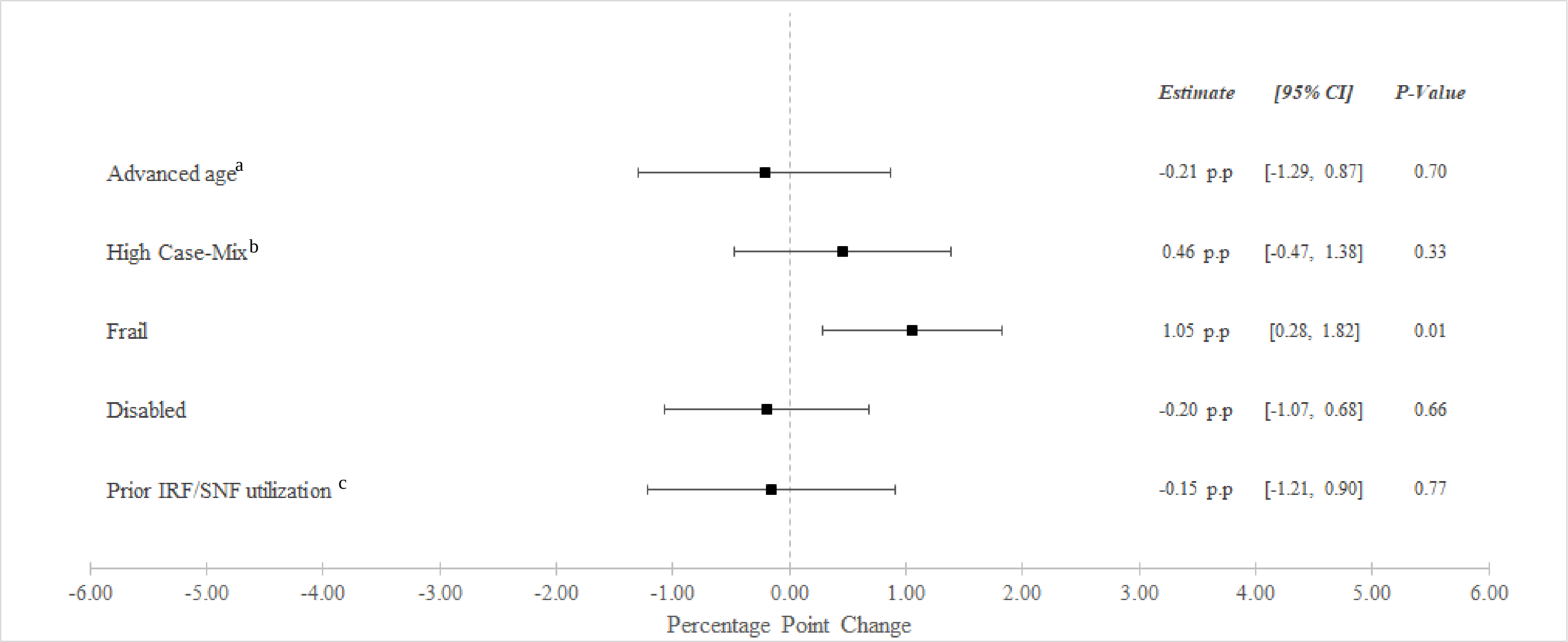

Abbreviations: IRF=Inpatient Rehabilitation Facility; SNF=Skilled Nursing Facility. [a]Age >85 years old. [b]Top 20% of elixhauser score. [c]Within preceding 12 months.

Figure S4. Adjusted Changes in Discharge Home with HHA for Medical Condition Episodes Among High-Risk Patients (2011-2016)

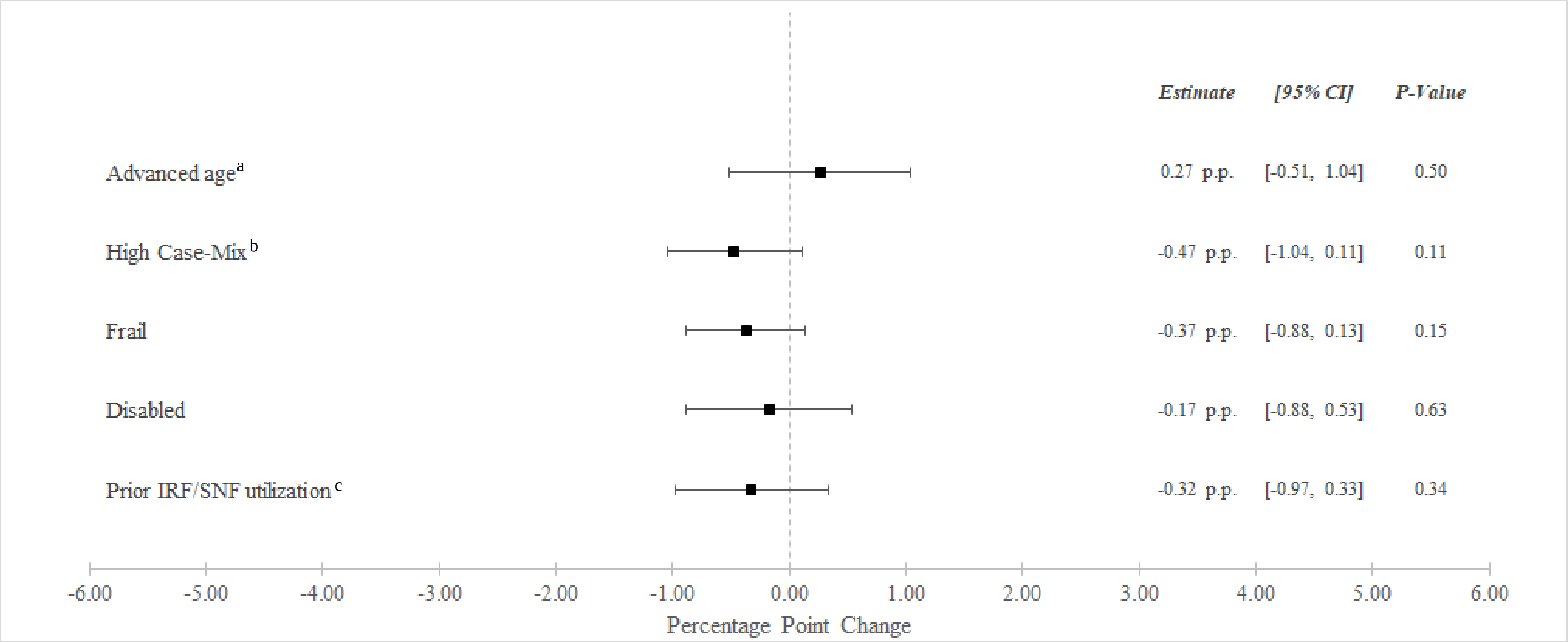

Abbreviations: HHA=Home Health Agency; IRF=Inpatient Rehabilitation Facility; SNF=Skilled Nursing Facility. [a]Age >85 years old. [b]Top 20% of elixhauser score. [c]Within preceding 12 months.

Figure S5. Adjusted Changes in SNF LOS for Medical Condition Episodes Among High-Risk Patients (2011-2016) Among Stayer-Hospitals Only

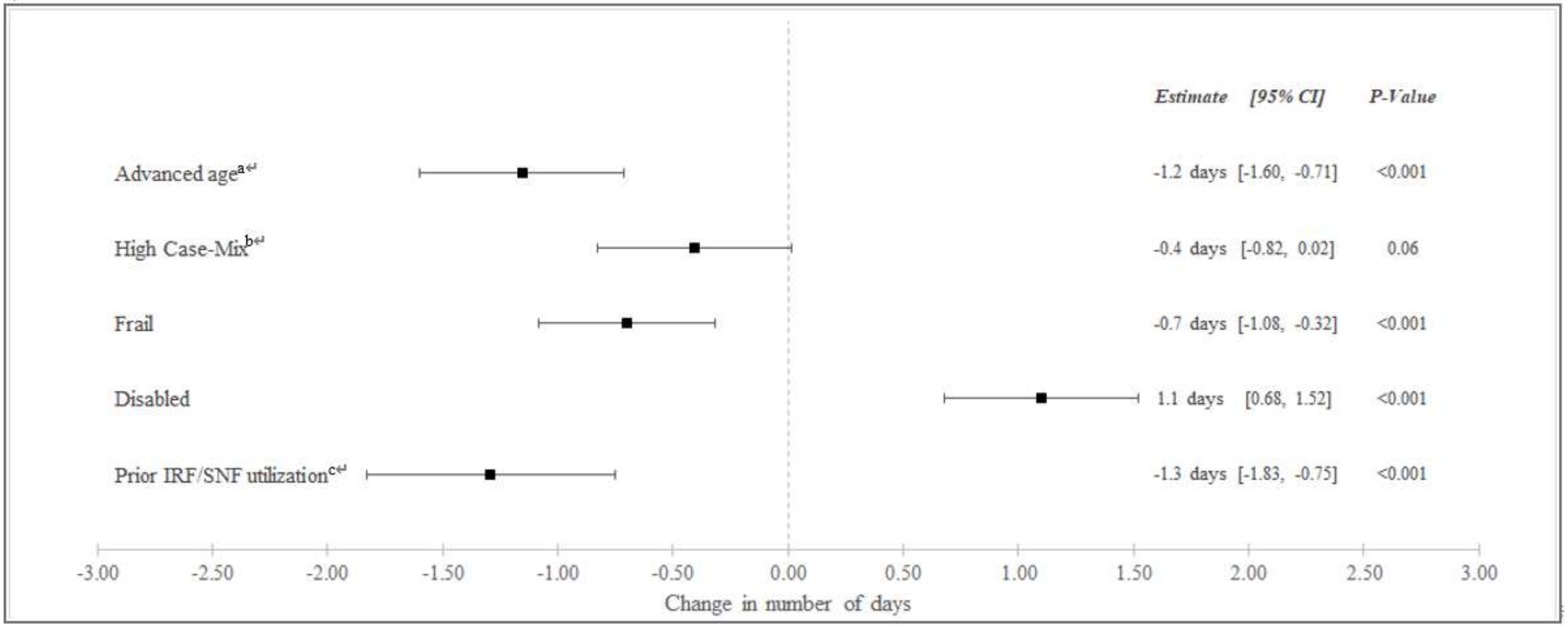

Abbreviations: LOS=length of stay; IRF=Inpatient Rehabilitation Facility; SNF=Skilled Nursing Facility. [a]Age >85 years old. [b]Top 20% of elixhauser score. [c]Within preceding 12 months.

Figure S6. Adjusted Changes in 90-Day Unplanned Readmission Rate‡ for Medical Condition Episodes Among High-Risk Patients (2011-2016) Among Stayer-Hospitals Only

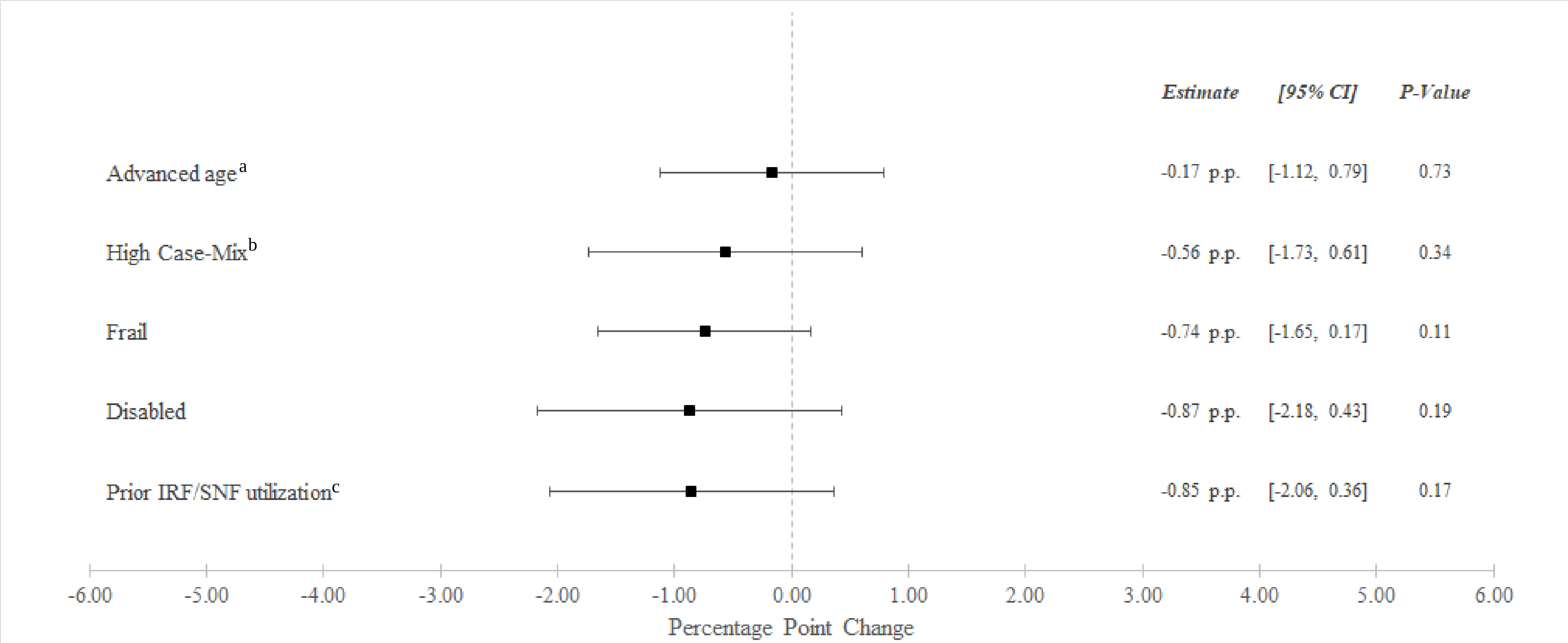

Abbreviations: IRF=Inpatient Rehabilitation Facility; SNF=Skilled Nursing Facility. ‡At least one acute care readmission during the 90-day episode. [a]Age >85 years old. [b]Top 20% of elixhauser score. [c]Within preceding 12 months.

Figure S7. Adjusted Changes in Post-Discharge Spending for Medical Condition Episodes Among High-Risk Patients (2011-2016) Among Stayer-Hospitals Only

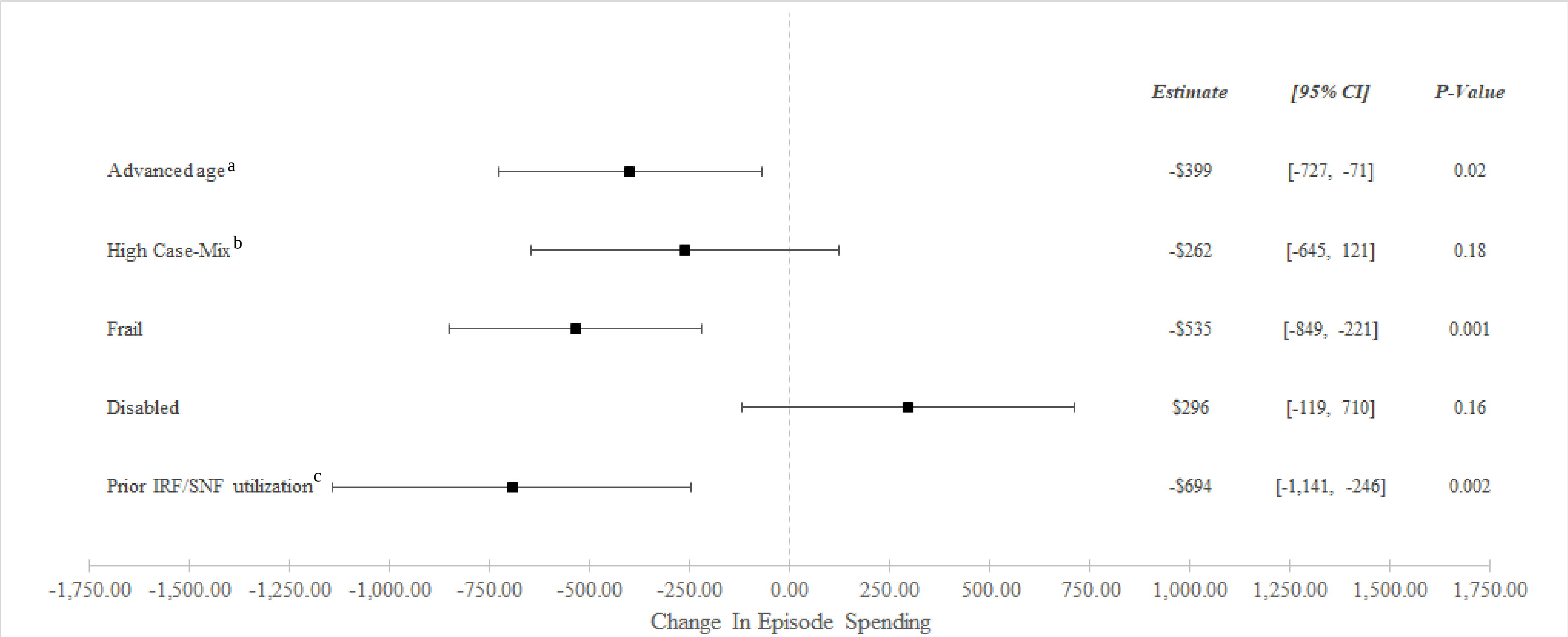

Abbreviations: IRF=Inpatient Rehabilitation Facility; SNF=Skilled Nursing Facility. [a]Age >85 years old. [b]Top 20% of elixhauser score. [c]Within preceding 12 months.

Figure S8. Adjusted Changes in 90-Day Mortality Rate† for Medical Condition Episodes Among High-Risk Patients (2011-2016) Among Stayer-Hospitals Only

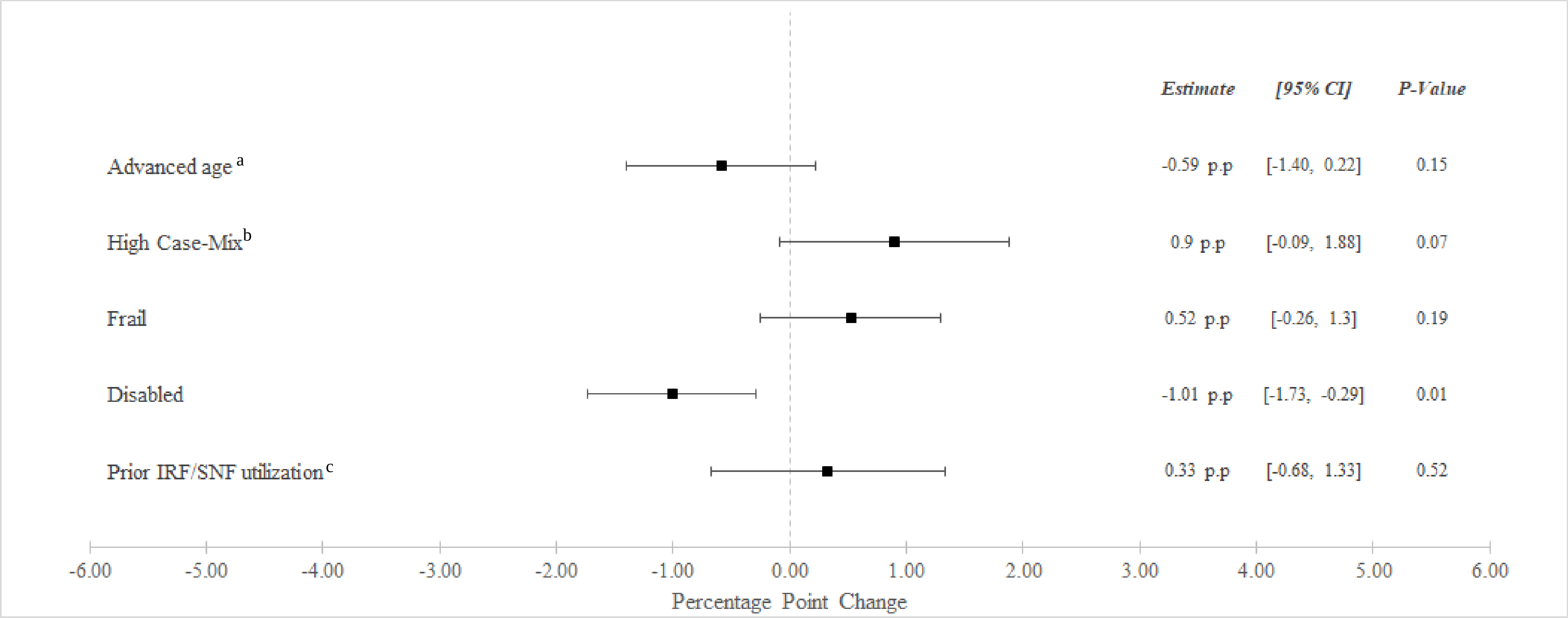

Abbreviations: IRF=Inpatient Rehabilitation Facility; SNF=Skilled Nursing Facility. †Percentage who died within 90 days after discharge alive from index hospitalization. [a]Age >85 years old. [b]Top 20% of elixhauser score. [c]Within preceding 12 months.

Figure S9. Adjusted Changes in Discharge to Institutional PAC Providers for Medical Condition Episodes Among High-Risk Patients (2011-2016) Among Stayer-Hospitals Only

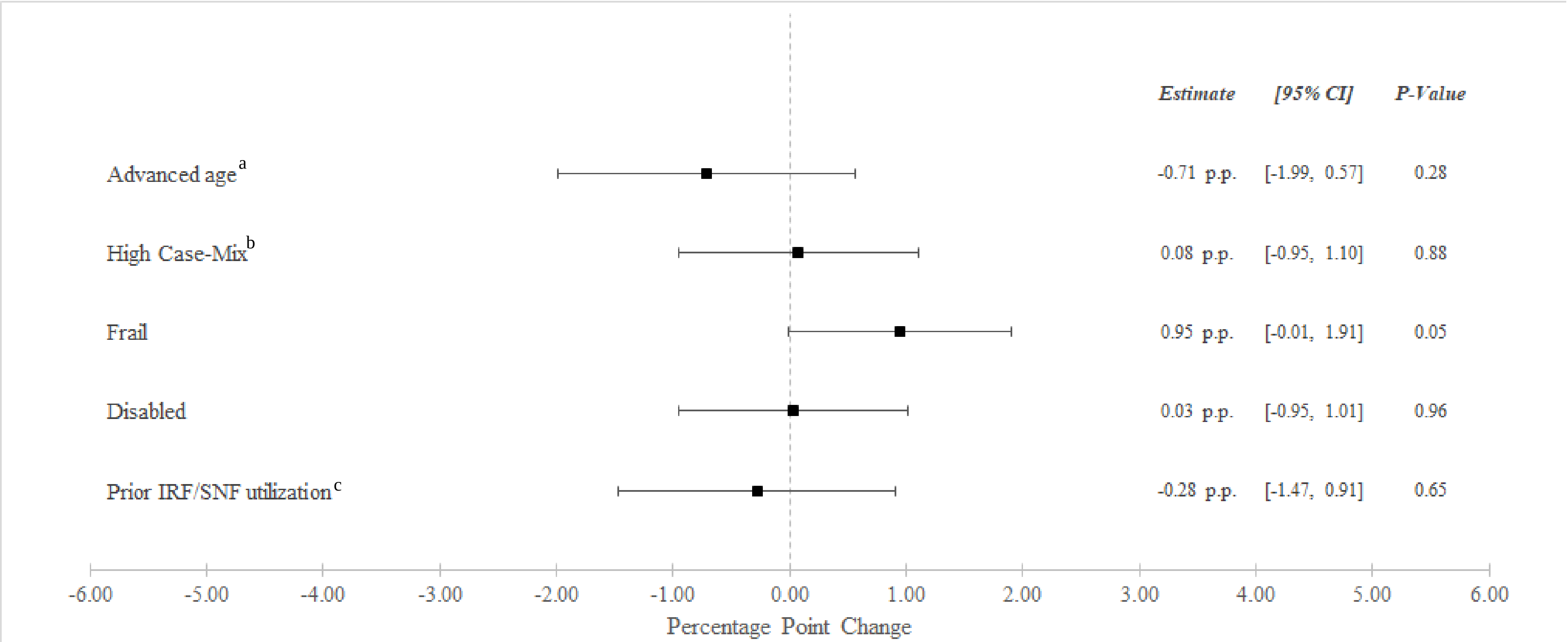

Abbreviations: IRF=Inpatient Rehabilitation Facility; SNF=Skilled Nursing Facility. [a]Age >85 years old. [b]Top 20% of elixhauser score. [c]Within preceding 12 months.

Figure S10. Adjusted Changes in Discharge Home with HHA for Medical Condition Episodes Among High-Risk Patients (2011-2016) Among Stayer-Hospitals Only

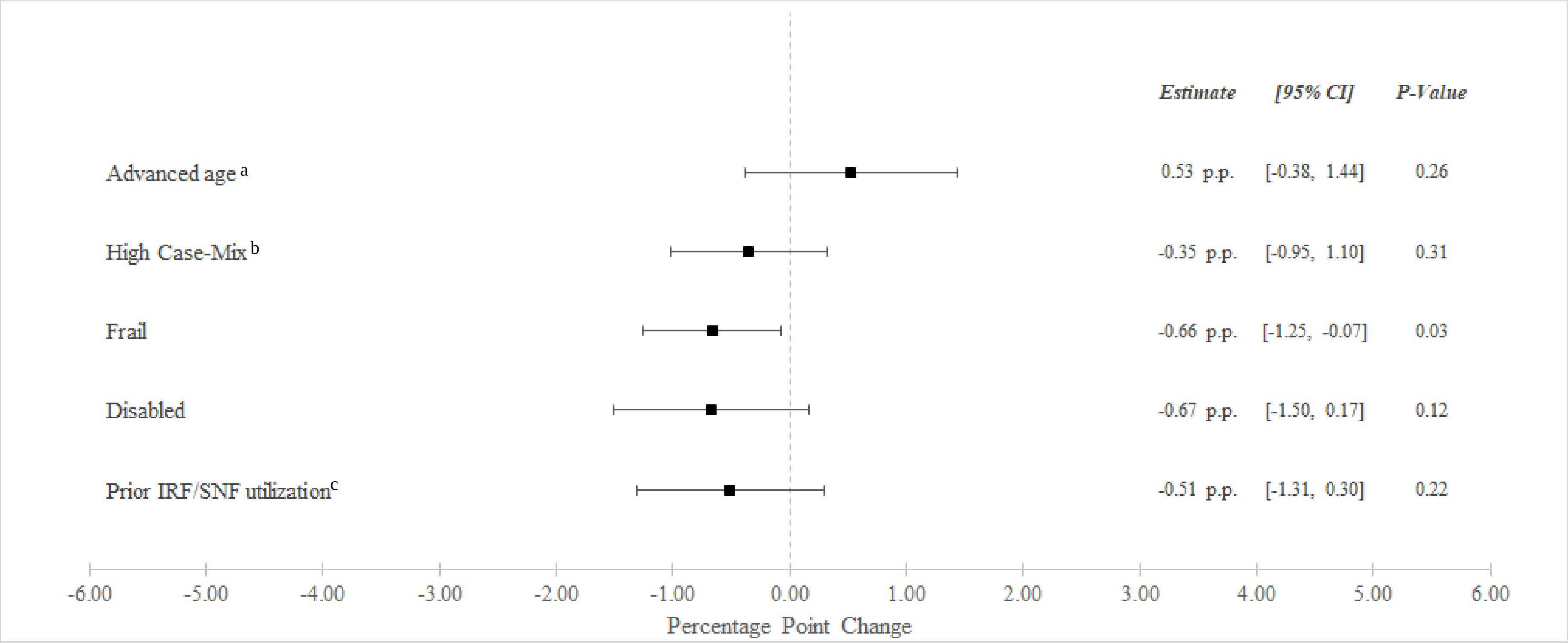

Abbreviations: HHA=Home Health Agency; IRF=Inpatient Rehabilitation Facility; SNF=Skilled Nursing Facility. [a]Age >85 years old. [b]Top 20% of elixhauser score. [c]Within preceding 12 months.
